# Heart transplantation after Fontan operation

**DOI:** 10.1016/j.xjtc.2022.01.020

**Published:** 2022-02-15

**Authors:** Igor E. Konstantinov, Antonia Schulz, Edward Buratto

**Affiliations:** a Department of Cardiothoracic Surgery, Royal Children's Hospital, Melbourne, Australia; b Department of Paediatrics, University of Melbourne, Melbourne, Australia; c Heart Research Group, Murdoch Children's Research Institute, Melbourne, Australia; d Melbourne Centre for Cardiovascular Genomics and Regenerative Medicine, Melbourne, Australia

**Keywords:** Fontan, single ventricle, transplantation

**Figure:**
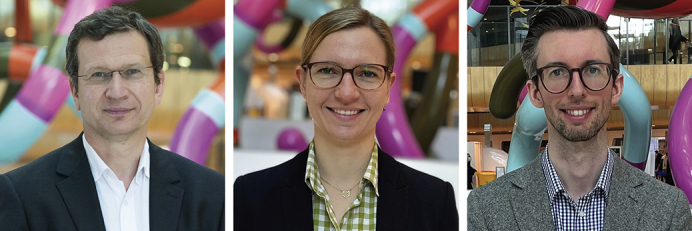
Drs Igor E. Konstantinov, Antonia Schulz, and Edward Buratto at the Royal Children's Hospital in Melbourne.

**Central Message**

The growing number of patients with failing Fontan circulation necessitates urgent development of a multidisciplinary model of expert care to address the perioperative complexity of heart transplantation.

See Commentary on page 192.

There is an increasing number of long-term survivors with complex congenital heart disease (CHD) and single ventricle palliation, adding to a growing cohort of patients with a Fontan circulation. In the modern era, survival at 20 years is greater than 90%.^1^ However, it appears that the fate of most patients who are palliated in this manner is to eventually develop failure of the single ventricle or the Fontan circulation. Currently, the ultimate treatment for the failed Fontan circulation is cardiac transplantation. Patients with failed Fontan circulation make up an increasing proportion of those presenting for heart transplantation.^2^ The need for expert surgical management of these complex patients with failing Fontan circulation has become increasingly clear during recent years.

## The Failing Fontan Circulation

**Figure 1:**
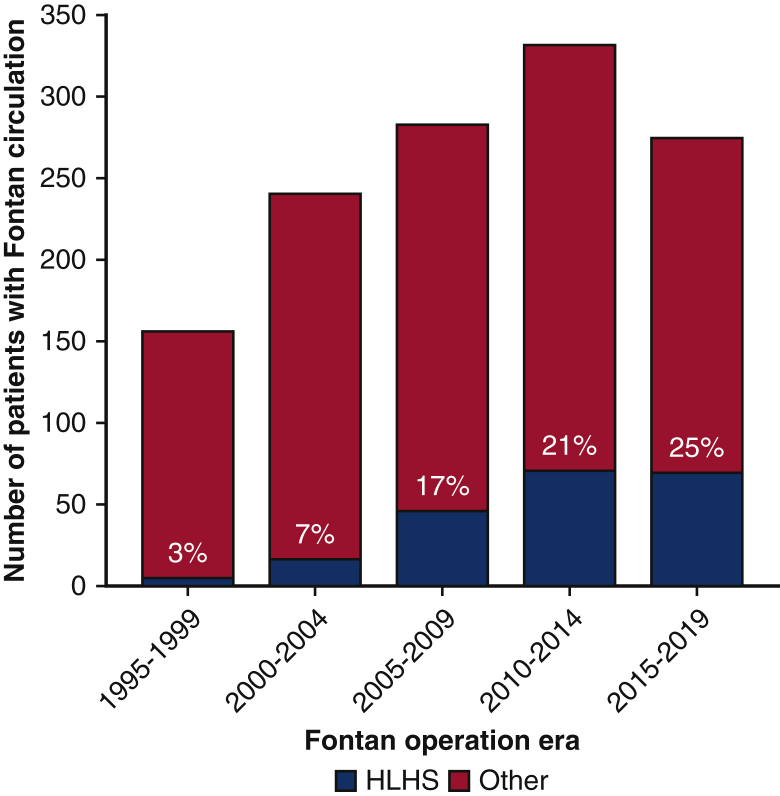
Increased proportion of patients with HLHS in 1290 survivors with Fontan circulation in Australia and New Zealand. *HLHS*, Hypoplastic left heart syndrome.

Overall, the incidence of Fontan failure is 26% to 30% at 20 years, and an underlying diagnosis of hypoplastic left heart syndrome (HLHS) has been identified as a primary risk factor.^1^^,^^3^ Failure of the Fontan circulation may occur in patients with impaired or preserved ventricular function.^4^, ^5^, ^6^ Of note, most of the patients with failed Fontan circulation have preserved ventricular function.^7^ As survival of the multistage palliation for HLHS continues to improve, the proportion of patients with Fontan circulation and HLHS is increasing. For instance, in 1290 patients in Australia and New Zealand who underwent the Fontan operation from 1995 to 2020, there was a dramatic proportional increase in patients with HLHS, reaching 25% for the most recent 5-year period (Figure 1). At some institutions, HLHS may be as prevalent as 50% in patients with Fontan circulation.^8^ It was previously demonstrated that right ventricular dominance is a risk factor for the failure of Fontan circulation.^9^ Thus, such an increase will inevitably lead to a higher proportion of patients having failure of the Fontan circulation, and subsequently being referred for transplantation. Most important, the technical complexity of transplantation in patients after Norwood-type aortic reconstruction is greater compared with that in other types of Fontan circulation.

## Perceived Inoperability and Inequality of Access to Heart Transplantation

Historically, patients with failing Fontan circulation were perceived to be of higher risk compared with other transplantation candidates with congenital heart disease.^10^, ^11^, ^12^ There are both real and perceived components to surgical complexity. Namely, transplantation after Fontan operation is more complex, particularly, in those with HLHS, yet the perceived impermissibly high risk of transplantation can be successfully mitigated in centers with specialized expertise. We^3^ and others^13^^,^^14^ have previously demonstrated that good outcomes can be achieved after heart transplantation in patients with Fontan failure. Yet, most patients with Fontan failure do not undergo heart transplantation because of the surgical complexity compounded by concerns of irreversible organ failure.

A need for multidisciplinary specialist care for the ever-growing population of patients with Fontan failure has become clear during recent years. An analysis of the Australia and New Zealand Fontan registry has demonstrated that only 2.5% of 1369 patients with Fontan circulation underwent transplantation at 20 years, whereas there was a higher proportion of patients with Fontan circulation failure.^3^ Furthermore, evaluation of referral patterns revealed that patients who were not treated at the national referral center were less likely to be considered for transplantation.^3^ This indicates the need for standardized guidelines for assessment and management for these challenging patients. Patients with failing Fontan circulation require early referral for transplant evaluation at a center of excellence where they can be assessed for all available treatment options. Most important, long-term survival after heart transplantation in Fontan patients is not different than that of patients with other forms of CHD.^3^ In short, no patient with failing Fontan circulation should be considered unfit for heart transplantation until appropriately assessed by the multidisciplinary group with combined expertise in surgical management of complex CHD, durable ventricular assist device (VAD) support, and heart transplantation.

## Candidacy for Transplantation

**Figure 2:**
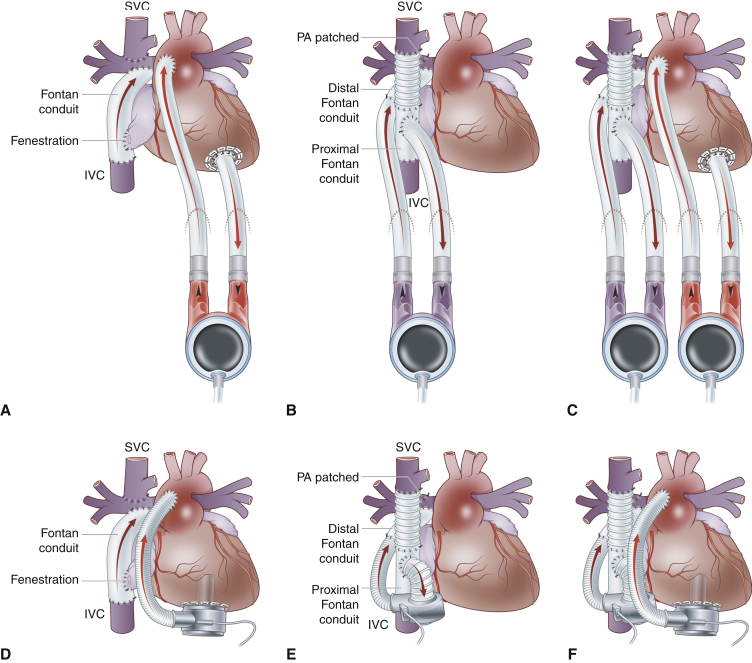
Durable VAD support in patients with Fontan circulation. Pulsatile VAD is used to support systemic (A) or pulmonary (B) circulation or both (C) in smaller children, whereas continuous flow VAD is used to support systemic (D) or pulmonary (E) circulation or both (F) in older children and adults. *SVC*, Superior vena cava; *IVC*, inferior vena cava; *PA*, pulmonary artery.

Perhaps the best way to determine if the multiorgan failure is reversible would be to support failing Fontan circulation with durable VAD, which could be of various configurations (Figure 2). Although most commonly one durable VAD is used to support systemic or pulmonary circulation, “biventricular” support can be used in rare cases, although the latter appears unnecessary in most patients. In many patients, end-organ dysfunction would be reversible with VAD support as a bridge to candidacy. Our group has previously demonstrated that the need for inotropic support and worsening renal function were predictors for death or delisting due to deterioration in children awaiting heart transplantation, yet they can be successfully bridged with VAD to heart transplantation.^15^ Needless to say, the configuration of VAD support must be tailored to the mode of Fontan failure and the procedure must be performed by a surgeon with expertise in managing patients with univentricular circulation.^16^, ^17^, ^18^ In particular, patients with preserved ventricular function often have a substantial burden of aortopulmonary collaterals and higher pulmonary vascular resistance, and appear to have increased mortality compared with patients with impaired function.^4^

Multiorgan dysfunction, especially renal and liver failure and plastic bronchitis, commonly occurs in the failing Fontan circulation. Although reasonable results have been reported for combined heart-liver transplantation,^19^ the indications for liver transplantation in Fontan-associated liver disease still need to be defined,^19^^,^^20^ and the benefit of such an approach remains unclear. However, with appropriate VAD support it is possible that improvement in liver function would occur in many patients, allowing heart transplantation to be performed in a more stable patient and minimizing the need for liver transplantation. Because this is a rapidly evolving field, there are still many unknowns, and currently there are limited data to support this notion. Likewise, heart-lung transplantation may be considered in patients with hypoplastic pulmonary arteries and pulmonary hypertension, yet a period of cavopulmonary VAD (Figure 2, *B* and *E*) support may result in significant improvement of pulmonary artery arborization and lowering of pulmonary resistance. The is exemplified by our recent Fontan patient with normal systolic function of the single ventricle and severe plastic bronchitis and protein-losing enteropathy. This 5-year-old girl could not be weaned off ventilatory support and deteriorated rapidly, requiring urgent extracorporeal membrane oxygenator support. In this state, the patient was not a candidate for heart transplantation. Cavopulmonary VAD insertion (Figure 2, *B*) resulted in complete resolution of protein-losing enteropathy and plastic bronchitis. She is has been supported by VAD for 588 days until she underwent successful heart transplantation. We and others have previously emphasized this approach as a bridge to candidacy for heart transplantation in patients with failing Fontan circulation and preserved single ventricle function.^16^^,^^21^^,^^22^ Yet, the current world experience is still limited to a handful of patients. Although a combined heart-lung-liver transplantation is technically feasible,^23^ the need for such procedure may be reduced in Fontan patients with appropriate VAD support.

## Technical Aspects of Transplantation after Fontan Operation

**Figure 3:**
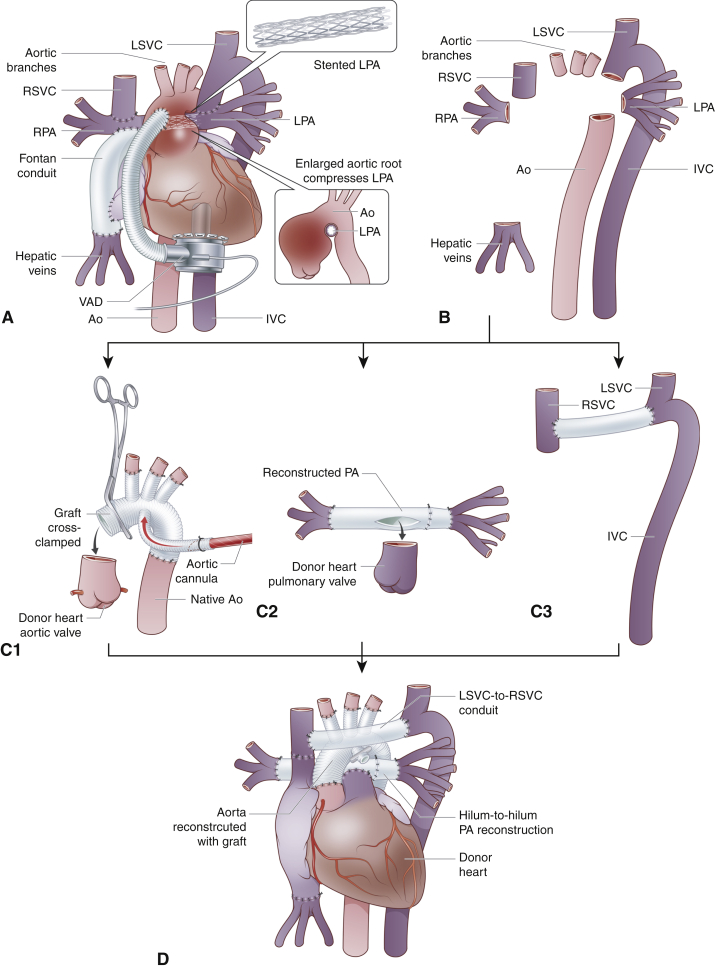
Complexity of surgical preparation for heart transplantation in patients with HLHS after Fontan operation. A, Enlarged aortic root after initial aortic reconstruction often compresses the pulmonary artery, and the latter may require stenting. Thus, anatomic separation of the aortic arch is often impossible, and the aortic arch replacement is required. B, In addition to reconstruction of aortic arch and central pulmonary arteries, patients with heterotaxy and anomalous systemic venous drainage require reconstruction of systemic venous pathways. C, Sequential reconstruction of aortic arch (C1), central pulmonary arteries (C2), and systemic venous drainage (C3) must be accomplished before arrival of the heart to minimize ischemic time of the donor heart. D, After heart transplantation, meticulous hemostasis is required to minimize bleeding from the numerous suture lines and ensure judicious use of blood products. *LSVC*, Left superior vena cava; *RSVC*, right superior vena cava; *RPA*, right pulmonary artery; *LPA*, left pulmonary artery; *VAD*, ventricular assist device; *Ao*, aorta; *IVC*, inferior vena cava.

We have previously reported that in patients undergoing heart transplantation after the Fontan in Australia and New Zealand, aortic arch reconstruction was required in 9% of patients and pulmonary artery reconstruction in 53%.^3^ As the proportion of Fontan patients with HLHS is steadily increasing, we expect that many more patients will require complex reconstruction of the aortic arch and pulmonary arteries.^24^^,^^25^ In fact, others have reported rates of up to 85.4% for pulmonary artery reconstruction.^12^ After Norwood-type reconstruction of the aortic root (Figure 3, *A*), the enlarged aortic root, the pulmonary artery, particularly after pulmonary artery stenting, and often the innominate vein are so densely adherent that they can only be removed en bloc, resulting in the need to reconstruct major vessels (Figure 3, *B*). This complexity can be compounded by heterotaxy syndromes, including azygos continuation of the inferior vena cava. Thus, it is crucial to achieve reconstruction of aortic arch (Figure 3, *C1*), pulmonary arteries (Figure 3, *C2*), and systemic venous drainage (Figure 3, *C3*) before arrival of the donor heart to minimize ischemic time. The systemic venous reconstruction can be achieved using donor innominate vein. Although it may appear tempting to use donor aorta and branch pulmonary arteries for reconstruction during heart transplantation, such temptation should be resisted, because it always takes longer than anticipated to reconstruct aorta and fragile pulmonary arteries, especially after stent removal. The pulmonary arteries and systemic veins can be reconstructed with prosthetic material or a homograft before donor heart arrival. Thus, the entire reconstruction is completed before transplantation (Figure 3, *D*).

**Figure 4:**
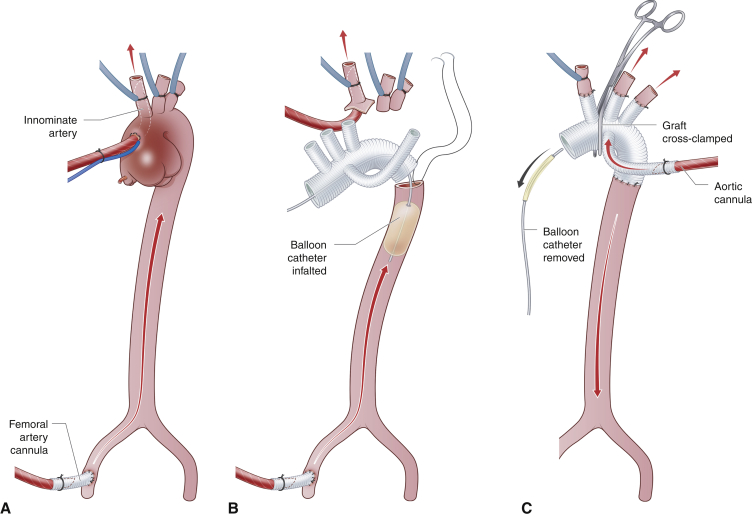
Aortic reconstruction after en bloc removal of the ascending aorta, aortic arch, and pulmonary arteries. A, A graft sutured to the femoral artery is cannulated. After hypothermia is achieved, the ascending aortic cannular is introduced into the innominate artery and the arch branches are snagged. B, A balloon catheter is introduced into the descending aorta via the graft so that a continuous lower body perfusion is maintained in addition to cerebral perfusion. C, Once left common carotid and left subclavian arteries are anastomosed, the balloon catheter is removed, the aorta is crossclamped between the carotid arteries and the antegrade perfusion of the whole body is reestablished. Once the innominate artery is reanastomosed, the aortic crossclamped is repositioned onto ascending aorta. If the time permit, while awaiting for the donor heart to arrive, the femoral artery can be reconstructed at this stage.

The aortic reconstruction must be planned in advance because this is the first crucial step of the vascular reconstruction before transplantation. Aortic arch reconstruction is often required after Norwood-type aortic repair and can be achieved using a vascular prosthesis.^24^, ^25^, ^26^, ^27^ We have previously reported a rate of 75% for reconstructions of the great vessels after previous univentricular palliation.^27^ In particular, in our experience, 50% of patients with prior Norwood-type reconstruction required aortic arch reconstruction.^27^ Reconstruction of the aortic arch should be performed before arrival of the donor heart to minimize ischemic time. This is achieved using hypothermia with continuous cerebral and lower body perfusion (Figure 4).

In contrast, transplantation in patients without prior Norwood-type aortic root reconstruction (eg, in those with tricuspid atresia) is easier because it does not usually require aortic arch replacement. In these patients, the use of donor central pulmonary arteries^25^ or donor descending aortic interposition graft for repair of pulmonary artery discontinuity can be considered.^28^ Likewise, a donor innominate vein can be used in this setting to reconstruct systemic venous return.

In patients with situs inversus or heterotaxia, more complex pretransplantation vascular reconstruction may result in significantly longer cardiopulmonary bypass times.^12^^,^^25^ In these patients, a graft may be used to reconstruct systemic venous return before the donor heart arrives. The technical aspects of heart transplantation in these patients may be daunting, and the plan for reconstruction must be considered before transplantation.

## Perioperative Management

Because of this technical complexity, it is crucial to have adequate preoperative planning and multidisciplinary team involvement to optimize outcomes in transplantation of patients with complex univentricular hearts.

A multidisciplinary team meeting should be held before surgery and include members from the surgical, cardiology, intensive care, and hematology teams. It is critical to plan for considerable bleeding after extensive dissection, especially in the context of prolonged bypass time and hypothermia. It is most crucial to ensure timely availability of blood products and other hemostatic adjuncts.

The timing of the recipient and donor procedures is most crucial to avoid unnecessary ischemic time for the donor heart. In our unit, patients are transferred to the operating room to allow 4 hours for surgical reconstruction before the donor heart arrival. Thus, precise coordination and close communication with the transplanting team are a must, because organ procurement must be delayed until the transplant team approves such procurement. The paramount importance of meticulous coordination between the transplant team and procurement team cannot be overemphasized. For patients with a bilateral superior vena cava, it is advantageous to bring a long segment of superior vena cava including the innominate vein because this facilitates reimplantation.^29^ However, in patients with azygos continuation of the inferior vena cava, an innominate vein reconstruction with a large graft before donor heart arrival may be required.

## Outcomes

Varying outcomes have been reported from single centers and larger databases after transplantation of this challenging group of patients. The recent studies are summarized in Table 1. There is a striking inconsistency in reporting the outcomes. Namely, some report the overall outcomes of transplantation in patients with CHD, mentioning the number of patients with Fontan circulation, yet do not mention the outcomes in the latter. Furthermore, even when the results are described specifically for Fontan patients, the outcomes of those with HLHS and others are not separated. The complexity for those who had Norwood-type reconstruction of the aorta is greater compared with those who did not, that is, in whom the ascending aorta could be crossclamped.

**Table 1:** Outcomes of heart transplantation in patients with Fontan circulation

First author	Publication year	Study time frame	Fontan patients	Age (y)	HLHS	Early mortality	Long-term survival
Gamba^32^	2004	1990-2002	14	17.2 ± 6.3	None	14.3%	86% (1 y) 77% (5 y) 62% (10 y)
Bernstein^30^	2006	1993-2001	70∗	10.7 ± 5.4	Not reported	Not reported	77% (1 y) 73% (3 y) 67% (5 y)
Kanter^13^	2011	1988-2010	27	8.2 ± 5.0	NA	3.7%	81.5% (1 y) 65.5% (5 y)
Voeller^2^	2012	1986-2009	34	Not reported for Fontan	Not reported for Fontan	23.5%	55.6% (10 y)
Davies^12^	2012	1984-2007	43	14.5 (1-47)	14.6%	25%	62.4% (1 y) 58.7% (5 y) 47.5% (10 y)
Karamlou^45^	2012	1993-2007	144†	25.4 ± 10.9	Not reported	23%	Not reported
Backer^46^	2013	1990-2012	22	14.9 ± 11.8	27.3%	23%	77% (1 y) 66% (5 y) 45% (10 y)
Iyengar^27^	2014	1988-2012	8	12.4 ± 6.1	50%	0%	Not reported for Fontan
Michielon^31^	2014	1991-2011	61‡	15 ± 9.7	26.2%	18.3%	81.9% (1 y) 73% (5 y) 56.8% (10 y)
Shi^3^	2016	1990-2015	34§	17 (11-31)	18%	5.8%	91% (1 y) 78% (5 y) 71% (10 y)
Murtuza^5^	2016	1990-2015	26	26.1 ± 8.6	None	30.8%,	65.4% (1 y)
						23.8% (recent era)	65.4% (1 y)
Miller^47^	2016	1995-2014	47	13 ± 6	Not reported	Not reported	early era: 63% (1 y) recent era:90% (1 y)
Tabarsi^33^	2017	1984-2013	351‖	14 (7-24)	18.3%	Not reported	80.3% (1 y) 71.2% (5 y)
Simpson^48^	2017	1993-2014	402¶	10.5 ± 4.9	Not reported	Not reported	recent era: 89% (1 y) 85% (3 y) 78% (5 y)
							early era: 77% (1 y) 74% (3 y) 72% (5 y) 64% (10 y) 42% (15 y)
Berg^34^	2017	1991-2014	36	21.3 (7.2-48.1)	28%	22.2%	75% (1 y) 58.3% (3 y)
Marrone^35^	2017	1987-2015	23	17.6 (11-33)	4.3%	17.4%	78% (1 y)
Serfas^14^	2020	2010-2018	537#	14 (10-18)	51%	7.6%	Not reported
Hernandez^36^	2020	2004-2014	93∗∗	24 (21-40)	Not reported	26.3%	Not reported
Riggs^49^	2021	2006-2017	130††	6 (4-11)	Not reported for Fontan patients	Not reported	all HLHS: 85% (1 y) 76% (5 y)
							Fontan: 85% (1 y) 80% (5 y)

*HLHS*, Hypoplastic left heart syndrome; *NA*, not available.

∗ Multi-Institutional Pediatric Heart Transplant Study.

† Nationwide Inpatient Sample data from the United States. This study included patients with single ventricle age 14 y and older. The authors were unable to determine whether all of those patients had undergone Fontan completion or remained in an intermediate palliated state.

‡ Multi-Institutional Study.

§ Australia and New Zealand Fontan Registry.

‖ Meta-analysis.

¶ Pediatric Heart Transplant Study database.

# Society of Thoracic Surgeons Congenital Heart Surgery Database.

∗∗ Nationwide Inpatient Sample database.

†† United Network for Organ Sharing database and Pediatric Health Information System.

The main causes of hospital mortality after heart transplantation are infection and graft failure.^30^^,^^31^ The long-term survival in the modern era appears to be good. For instance, patients from the Australian and New Zealand Fontan registry who underwent transplantation between 1990 and 2015 (n = 34) had a post-transplant survival of 91%, 78%, and 71% at 1, 5, and 10 years, respectively.^3^ These patients had similar long-term survival to other patients with CHD undergoing heart transplantation.^3^ We have previously demonstrated that patients with CHD had a higher 30-day mortality compared with patients without CHD, but similar survival after 10 years.^25^

Multiple studies have reported an early mortality ranging from 14% to 30%.^2^^,^^5^^,^^12^^,^^31^, ^32^, ^33^, ^34^, ^35^, ^36^ Yet, we^3^^,^^27^ and others^13^ have demonstrated that an early mortality of 0% to 5.8%, and good long-term survival can be achieved in specialized institutions with appropriate expertise. A recent multi-institutional study that included 51% of patients with HLHS demonstrated an early mortality of 7.6%.^14^ Thus, there is mounting evidence to suggest that low operative mortality and good long-term results can be achieved in these challenging patients with a focused approach.

## Indiscriminate Reporting of the Outcomes of Heart Transplantation after Fontan Operation

It is fascinating that the latest version of the STAT Mortality Scores and Categories assigns heart transplantation, regardless of the status of the recipient (ie, univentricular or biventricular circulation, primary or redo), to the STAT 3 category.^37^ Addition of pulmonary artery reconstruction would put these patients into the STAT 4 category. However, the additional risk of aortic arch replacement after Norwood-type reconstruction is not accounted for, although it is likely that this would further contribute to complexity and risk. Heart transplantation as a primary procedure in a patient without any prior heart surgery is one of the easiest cardiac operations. Clearly, the complexity and the risk of heart transplantation would be very different in patients without prior heart surgery compared with heart transplantation after the Fontan operation in patients with HLHS. Moreover, even in patients with Fontan circulation, the complexity of heart transplantation in those with and without prior aortic arch reconstruction is considerably different. Therefore, the operative mortality after heart transplantation in Fontan patients with and without prior aortic arch reconstruction should be reported separately to allow for appropriate risk stratification.

## Access to Transplantation

An analysis of the Australia and New Zealand Fontan registry has shown that 2.5% of patients with Fontan circulation underwent transplantation at 20 years, whereas there was a higher rate of patients with Fontan failure.^3^^,^^38^ Furthermore, evaluation of referral patterns revealed that patients who were not followed up at the national referral center were less likely to be considered for transplantation.^3^ Patients with failing Fontan circulation require early referral for transplant evaluation to a center of excellence where they can be assessed for all available surgical treatment options and listed in time for transplantation, before becoming high-risk candidates. We have previously demonstrated the cost-effectiveness of the centralized national pediatric heart transplantation program in Australia.^39^ Although direct comparison is difficult, the study suggested that the cost-utility of pediatric heart transplantation in Australia appeared more favorable than that reported in the United States.^39^ Furthermore, the positive relationship between increasing operative volume and improved outcomes in heart transplantation has been documented.^40^, ^41^, ^42^ In particular, an increased mortality has been observed for centers performing fewer than 10 to 14 heart transplantations per year.^40^, ^41^, ^42^ In the United States, a theoretical regionalization simulation appears to be associated with improved mortality after heart transplantation.^43^ Although there are currently no data available to support this notion, one would expect that such reduction in mortality can only be amplified in high-risk patients with failing Fontan circulation. It should also be remembered that a low-volume center, without highly specialized expertise in congenital heart disease, patients with failing complex Fontan circulation may be labeled as inoperable, limiting their access to heart transplantation. Needless to say, regionalization is a complex issue,^44^ yet traveling to a higher-volume center to improve survival sounds like a small price to pay.

## Conclusions

Heart transplantation in the ever-increasing population of patients with failed Fontan circulation is a complex issue. It appears that many patients with failed Fontan circulation may be denied heart transplantation because of a perceived impermissibly high risk of surgery due to its complexity and apparent multiorgan failure. Current literature is lacking risk-stratified reporting of the outcomes of heart transplant recipients with failing Fontan circulation.

### Conflict of Interest Statement

The authors reported no conflicts of interest.

The *Journal* policy requires editors and reviewers to disclose conflicts of interest and to decline handling or reviewing manuscripts for which they may have a conflict of interest. The editors and reviewers of this article have no conflicts of interest.

## Figures and Tables

**Table:** 

	**JTCVS**: Protein-losing enteropathy and plastic bronchitis after the Fontan procedure. *Sharma VJ, Iyengar AJ, Zannino D, Gentles T, Justo R, Celermajer DS, Bullock A, Winlaw D, Wheaton G, Burchill L, Cordina R, d'Udekem Y.* J Thorac Cardiovasc Surg*.* 2021;161(6):2158-2165.
	**Commentary**: Late adverse outcomes of the Fontan circulation: A PLEa for standardization of diagnostic criteria. *Lubert AM, Redington AN.* J Thorac Cardiovasc Surg*.* 2021;161(6):2167-2168.
	**Commentary**: Lymphatic fluid flows uphill in both hemispheres-and leaks in the same places. *Woods RK, Hraska V.* J Thorac Cardiovasc Surg*.* 2021;161(6):2166-2167.
	**JTCVS**: Cost-effectiveness of the National Pediatric Heart Transplantation Program in Australia. *Ye XT, Parker A, Brink J, Weintraub RG, Konstantinov IE.* J Thorac Cardiovasc Surg*.* 2019;157(3):1158-1166.
	**Commentary**: Buy or sell: Pediatric cardiac transplantation is still a societal bull market…for now. *Evers PD, Morales D.* J Thorac Cardiovasc Surg*.* 2019;157(3):1167.
	**JTCVS**: Regionalization for thoracic surgery: Economic implications of regionalization in the United States. *Subramanian MP, Yang Z, Chang SH, Puri V.* J Thorac Cardiovasc Surg*.* 2021;161(5):1705-1709.
	**Commentary**: Go big or stay home? *Chilakamarry S, Boffa DJ.* J Thorac Cardiovasc Surg. 2021;161(5):1711-1712.
	**Commentary**: Regionalization for every region? *Fernandez FG.* J Thorac Cardiovasc Surg*.* 2021;161(5):1710.
